# Integrated Network Pharmacology and Molecular Dynamics Reveal Multi-Target Anticancer Mechanisms of *Myrtus communis* Essential Oils

**DOI:** 10.3390/ph18101542

**Published:** 2025-10-13

**Authors:** Ahmed Bayoudh, Nidhal Tarhouni, Riadh Ben Mansour, Saoussen Mekrazi, Raoudha Sadraoui, Karim Kriaa, Zakarya Ahmed, Ahlem Soussi, Imen Kallel, Bilel Hadrich

**Affiliations:** 1 Laboratory of Enzyme Engineering and Microbiology, Engineering National School of Sfax (ENIS), University of Sfax, P.O. Box 1173, Sfax 3038, Tunisia; ahmed.bayoudh@fss.usf.tn (A.B.); nidhal.tarhouni@estudiante.uam.es (N.T.); 2 Laboratory of Molecular and Functional Genetics, Faculty of Science, University of Sfax, Sfax 3038, Tunisia; riadh.benmansour@fss.usf.tn; 3 Research Laboratory of Environmental Toxicology-Microbiology and Health (LR17ES06), Faculty of Sciences, University of Sfax, Sfax 3038, Tunisia; mekrazisaoussen@gmail.com (S.M.); kallel1imen@yahoo.fr (I.K.); 4 Laboratory of Biotechnology and Biomonitoring of the Environment and Oasis Ecosystems (LBBEEO), Faculty of Sciences of Gafsa, University of Gafsa, Gafsa 2112, Tunisia; sadraouiraoudha@yahoo.fr; 5 Department of Chemical Engineering, College of Engineering, Imam Mohammad Ibn Saud Islamic University (IMSIU), Riyadh 11432, Saudi Arabia; kskriaa@imamu.edu.sa (K.K.); zalahmed@imamu.edu.sa (Z.A.); 6 Laboratory of Environmental Physiopathology, Valorization of Bioactive Molecules and Mathematical Modeling, Faculty of Sciences, University of Sfax, Sfax 3038, Tunisia; ahlem.soussi@fss.usf.tn

**Keywords:** *Myrtus communis*, essential oils, anticancer, network pharmacology, molecular dynamics, spathulenol, androgen receptor

## Abstract

**Background:** Cancer’s multifactorial complexity demands innovative polypharmacological strategies that can simultaneously target multiple oncogenic pathways. Natural products, with their inherent chemical diversity, offer promising multi-target therapeutic potential. This study comprehensively investigates the anticancer mechanisms of Tunisian *Myrtus communis* essential oils (McEOs) using an integrated computational-experimental framework to elucidate their polypharmacological basis and therapeutic potential. **Methods:** McEO composition was characterized via GC-MS analysis. Antiproliferative activity was evaluated against HeLa (cervical), MCF-7 (breast), and Raji (lymphoma) cancer cell lines using MTT assays. A multi-scale computational pipeline integrated network pharmacology, molecular docking against eight key oncoproteins, and 100 ns all-atom molecular dynamics simulations to elucidate molecular mechanisms and target interactions. **Results:** GC-MS revealed a 1,8-cineole-rich chemotype (38.94%) containing significant sesquiterpenes. McEO demonstrated potent differential cytotoxicity: HeLa (IC_50_ = 8.12 μg/mL) > MCF-7 (IC_50_ = 19.59 μg/mL) > Raji cells (IC_50_ = 27.32 μg/mL). Network pharmacology quantitatively explained this differential sensitivity through target overlap analysis, showing higher associations with breast (23%) and cervical (18.3%) versus lymphoma (5.5%) cancer pathways. Molecular docking identified spathulenol as a high-affinity Androgen Receptor (AR) antagonist (XP GScore: −9.650 kcal/mol). Molecular dynamics simulations confirmed exceptional spathulenol-AR complex stability, maintaining critical hydrogen bonding with Asn705 for 96% of simulation time. **Conclusions:** McEO exerts sophisticated multi-target anticancer effects through synergistic constituent interactions, notably spathulenol’s potent AR antagonism. This integrated computational-experimental approach validates McEO’s polypharmacological basis and supports its therapeutic potential, particularly for hormone-dependent malignancies, while establishing a robust framework for natural product bioactivity deconvolution.

## 1. Introduction

Cancer continues to represent one of the most pressing global health challenges of the 21st century. The International Agency for Research on Cancer reported approximately 20 million new cases and 9.7 million deaths worldwide in 2022, with projections indicating the number of new cancer cases per year could rise to 35 million by 2050 [1]. The prevailing “one-drug, one-target” paradigm in cancer drug development has demonstrated significant limitations. Clinical development success rates for oncology compounds remain challenging at approximately 10.4% from first-in-human trials to approval, compared to 15% across all therapeutic areas [2]. This low success rate reflects a fundamental disconnect between traditional reductionist therapeutic strategies and the complex, interconnected molecular networks that characterize cancer pathogenesis [3]. The intrinsic heterogeneity of cancer, involving multiple dysregulated pathways and adaptive resistance mechanisms, necessitates a paradigm shift toward polypharmacological approaches that can simultaneously modulate multiple therapeutic targets [4,5].

Natural products have historically served as invaluable sources of anticancer therapeutics, contributing to approximately 60% of currently approved oncological agents [6,7]. This remarkable success stems from the evolutionary optimization of natural compounds for high-affinity interactions with biological macromolecules, often resulting in unique mechanisms of action that complement synthetic drug discovery efforts. Recent advances in natural product research have highlighted their continued importance, with over 70% of approved anticancer drugs being natural products or their derivatives [8,9]. The inherent structural diversity and polypharmacological properties of natural products make them particularly well-suited for addressing the multi-target nature of cancer, offering therapeutic advantages that single synthetic compounds often cannot achieve.

Among natural products, essential oils represent a particularly compelling class of bioactive compounds characterized by complex mixtures of volatile phytochemicals that function as integrated therapeutic systems [10,11,12]. Unlike single-component drugs, essential oils embody the principles of polypharmacology through their inherent multi-component nature, where synergistic interactions among diverse constituents can modulate networks of molecular targets simultaneously [13,14]. This polypharmacological profile aligns with a contemporary understanding of cancer as a systems-level disease requiring multi-target therapeutic intervention [15]. Modern research has increasingly recognized the potential of essential oils in cancer therapy, with numerous studies demonstrating their ability to induce apoptosis, inhibit cell proliferation, and overcome drug resistance mechanisms [10,13].

*Myrtus communis* is a Mediterranean evergreen shrub with extensive traditional medicinal use spanning millennia. Ethnopharmacological applications have focused on antiseptic, anti-inflammatory, and antimicrobial properties across Mediterranean traditional medicine systems [16,17]. Modern phytochemical investigations have identified oxygenated monoterpenes as predominant bioactive constituents, with 1,8-cineole and linalool as major components alongside β-fenchol and methyleugenol [18]. Emerging preclinical evidence suggests that *Myrtus communis* essential oil (McEO) possesses promising anticancer properties. Recent studies have demonstrated dose-dependent cytotoxicity against various cancer cell lines, with McEO reducing viability in prostate cancer cells (IC_50_: 100–400 µg/mL), activating caspase-3 and PARP cleavage, and impairing cell migration [19].

Despite these promising initial observations, significant knowledge gaps persist in our understanding of McEO’s anticancer mechanisms. Current research lacks comprehensive standardized compositional analysis, limiting the ability to establish structure-activity relationships and ensure reproducibility across studies. More critically, the polypharmacological landscape of McEO as a complete therapeutic system remains largely unexplored. The potential for synergistic interactions among individual constituents, the full spectrum of molecular targets modulated by the essential oil, and the mechanistic basis for observed anticancer activities require systematic investigation using contemporary analytical and computational approaches.

The complexity of essential oil compositions and their biological effects necessitate integrative research methodologies that can bridge chemical characterization with mechanistic understanding. Network pharmacology, combined with molecular docking and molecular dynamics simulations, offers powerful tools for elucidating multi-target mechanisms and predicting drug-target interactions in complex natural product mixtures [20,21]. This computational framework, when integrated with experimental validation, can provide unprecedented insights into the molecular basis of essential oil bioactivity by mapping compound-target networks and identifying key therapeutic pathways [5,22]. Recent applications of network pharmacology to natural products have successfully revealed previously unknown mechanisms of action and identified novel therapeutic targets [23,24].

To address these critical knowledge gaps and test our hypothesis, this study employs a comprehensive multidisciplinary approach encompassing three primary objectives: detailed phytochemical characterization through gas chromatography-mass spectrometry analysis coupled with antioxidant capacity evaluation to establish a standardized compositional profile; in vitro systematic assessment of antiproliferative effects against representative cancer cell lines including HeLa, MCF-7, and Raji to quantify biological activities across different cancer types; and computational mechanistic elucidation through integrated network pharmacology, molecular docking, and extended molecular dynamics simulations to reveal the molecular mechanisms underlying the observed anticancer activities.

This integrative research strategy may provide a robust mechanistic framework that bridges chemical composition with biological function, combining rigorous experimental validation with state-of-the-art computational modeling. The outcomes of this investigation will advance our understanding of McEO’s anticancer potential, identify key bioactive compounds and their molecular targets, and elucidate the mechanistic basis for observed biological activities. Furthermore, this work will contribute to the broader development of natural product-based polypharmacological approaches for cancer treatment, potentially informing future therapeutic strategies that harness the inherent complexity of natural systems for improved clinical outcomes.

## 2. Results and Discussion

### 2.1. Chemical Composition and Phytochemical Profile of McEO

Gas chromatography-mass spectrometry (GC-MS) analysis successfully identified and quantified 16 primary compounds in McEO, collectively accounting for 98.03% of the total oil composition (Table 1; Figure S1). The chemical profile was characterized by a predominance of oxygenated monoterpenes, followed by sesquiterpenes and phenylpropanoids, reflecting the typical terpenoid-rich nature of Mediterranean *Myrtaceae* essential oils [25]. The essential oil yield obtained from 100 g of dried leaves was 0.87% (*w*/*w*). This robust yield surpasses those documented in several other Tunisian studies, which report yields in the range of 0.28–0.53% [26], and is highly comparable to the 0.9% yield documented in neighboring Algeria [27].

### 2.2. Antioxidant Activity

The antioxidant profile of McEO revealed significant variability across mechanistic assays, reflecting its chemotype-dependent bioactivity. Comprehensive evaluation using six complementary methods (DPPH, ABTS, FRAP, H_2_O_2_, O_2_^−^, and NO assays) demonstrated IC_50_ values spanning 0.008–1.482 mg/mL (Table 2, Figure S2). This heterogeneity underscores the necessity of multi-assay frameworks for evaluating complex natural products [28].

McEO demonstrated exceptional ferric-reducing capacity in the FRAP assay (IC_50_ = 0.008 ± 0.001 mg/mL), suggesting high ET capability, likely due to its content of oxygenated monoterpenes such as 1,8-cineole and α-terpineol, consistent with previously characterized Tunisian chemotypes [18]. The DPPH radical scavenging assay revealed moderate activity (IC_50_ = 0.138 ± 0.112 mg/mL), aligning with values reported by [29], where hydrodistilled McEO showed IC_50_ ≈ 941 µg/mL. In contrast, Snoussi et al. [18] found enhanced activity in floral bud-derived EO (IC_50_ = 240 µg/mL), emphasizing organ-dependent differences in antioxidant efficiency. The ABTS assay supported this trend, with McEO IC_50_ = 0.505 ± 0.050 mg/mL—moderate in comparison to ethanol extracts of *M. communis*, which often display stronger polar radical scavenging due to higher phenolic content [30]. Mechanism-specific assays showed relatively weak activity against ROS and RNS: IC_50_ = 0.555 ± 0.055 mg/mL (H_2_O_2_), 0.582 ± 0.058 mg/mL (NO), and 1.482 ± 0.148 mg/mL (O_2_^−^). These results indicate limited efficacy in hydrogen atom transfer (HAT) or direct radical quenching pathways, corroborating findings from comparative organ-based studies [18]. The observed antioxidant heterogeneity may arise from synergistic or antagonistic interactions among McEO constituents. While monoterpene like 1,8-cineole is abundant and known for moderate antioxidant effects, correlation analyses suggest that sesquiterpenes, although present in smaller amounts, contribute significantly to radical scavenging via synergistic interactions [31]. Overall, McEO demonstrates a selective antioxidant signature characterized by strong electron-donating (FRAP) capacity but moderate-to-low radical scavenging via HAT-based mechanisms. These results underscore the importance of chemotypic profiling, organ selection, and multi-assay evaluation when characterizing essential oils for functional or therapeutic applications.

### 2.3. Antiproliferative Effects

**Figure 1:**
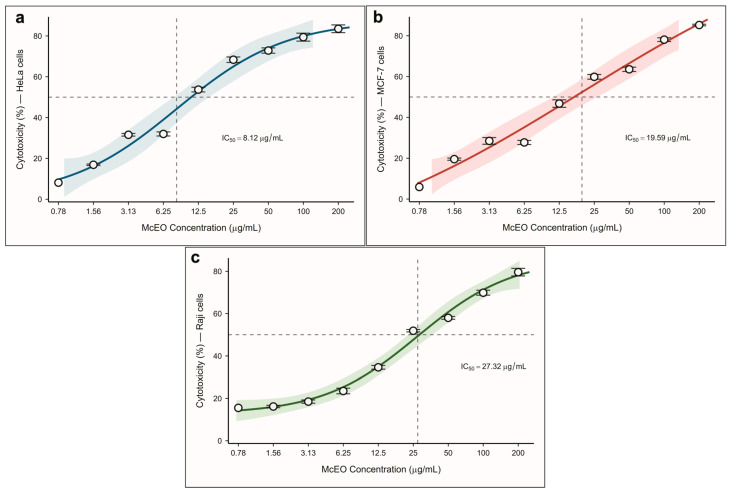
Dose–response curves of McEO cytotoxicity against different cancer cell lines. Antiproliferative effects of McEO against human cancer cell lines. Dose–response curves showing cytotoxicity percentage versus McEO concentration for (**a**) HeLa cells, (**b**) MCF-7 cells, and (**c**) Raji cells. Data points represent mean ± standard deviation (n = 3). The horizontal dashed line indicates 50% cytotoxicity, and vertical dashed lines show the calculated IC_50_ values for each cell line. Curves were fitted using four-parameter log-logistic nonlinear regression analysis. IC_50_ values: HeLa = 8.12 μg/mL, MCF-7 = 19.59 μg/mL, Raji = 27.32 μg/mL.

The antiproliferative activity of McEO was evaluated against three distinct cancer cell lines representing different tumor types and origins (Figure 1a,b, Table 3). The results revealed marked differences in cellular sensitivity, with IC_50_ values ranging from 8.12 to 27.32 μg/mL, demonstrating the selective cytotoxic potential of our essential oil preparation.

HeLa cells demonstrated exceptional susceptibility to McEO treatment, exhibiting the lowest IC_50_ value of 8.12 μg/mL among all tested cell lines (Figure 1a, Table 3). This pronounced sensitivity positions our McEO among the most potent natural anticancer agents reported for cervical cancer cells. Our IC_50_ of 8.12 μg/mL represents remarkable potency compared to other *Myrtus communis* preparations reported in the literature. This value demonstrates a 4-fold improvement over *M. communis* leaf extracts (IC_50_ = 33.3 μg/mL) [32] and superior activity compared to Serbian *M. communis* essential oil (IC_50_ = 15.2 μg/mL) [33]. Notably, our results are comparable to highly potent essential oils such as *Cinnamomum zeylanicum* (IC_50_ = 0.13 μg/mL) [34] and *Lawsonia inermis* (IC_50_ = 1.43 μg/mL) [35] positioning McEO as a promising therapeutic candidate. The superior potency of our McEO against HeLa cells appears to align with the established sensitivity of cervical cancer cells to terpenoid-rich essential oils. *Juniperus communis* essential oil demonstrated similar selectivity for HeLa cells (IC_50_ = 10.14 μg/mL) [36], with mechanisms involving caspase-3 upregulation and mitochondrial membrane depolarization. The rapid onset kinetics observed in our study suggest that McEO may effectively target multiple cellular pathways essential for cancer cell survival, potentially through similar apoptotic mechanisms involving Bax/Bcl-2 ratio modulation, as reported for other *Myrtus* species [37].

**Table 3:** IC_50_ values of McEO and doxorubicin against human cancer cell lines.

Cell Line	McEO (IC_50_, µg/mL)	Doxorubicin (IC_50_, µg/mL)
HeLa	8.12 ± 0.54	1.11 ± 0.10 [38]
MCF-7	19.59 ± 1.02	1.16 ± 0.03 [39]
Raji	27.32 ± 1.65	1.36 ± 0.04 [40]

IC_50_ values represent the concentration required to inhibit 50% of cell viability. Data are presented as mean ± standard deviation from three independent experiments. Doxorubicin IC_50_ values were converted from literature-reported µM values using the molecular weight of doxorubicin hydrochloride (579.99 g/mol).

MCF-7 cells demonstrated intermediate sensitivity with an IC_50_ of 19.59 μg/mL (Figure 1b, Table 3), representing a 2.4-fold reduction in potency compared to HeLa cells. The dose–response curve exhibited a relatively linear pattern across the tested concentration range, with steady cytotoxic progression from low to high concentrations. This IC_50_ value shows a 2.1-fold improvement over *M. communis* leaf extracts (IC_50_ = 41.5 μg/mL) [32] and substantially outperforms other geographic variants (IC_50_ = 597–762 μg/mL) [41]. The linear dose–response profile suggests a potentially consistent mechanism of action across the concentration range tested. Unlike the sigmoidal curve observed in HeLa cells, MCF-7 cells appeared to show a more gradual cytotoxic response, which may indicate different cellular vulnerability or resistance mechanisms. This pattern could be consistent with the hormone receptor-positive nature of MCF-7 cells, which often exhibit more gradual responses to cytotoxic agents compared to highly proliferative cell lines. The moderate sensitivity of MCF-7 cells to McEO may have important therapeutic implications for estrogen receptor-positive breast cancers. The relatively linear dose–response relationship suggests potentially predictable cytotoxic effects, which could facilitate dosing optimization in therapeutic applications. Furthermore, the intermediate potency might offer advantages in combination therapies, where McEO could potentially provide sustained cytotoxic pressure while minimizing off-target effects on normal tissues.

Raji cells exhibited the highest resistance among the tested cell lines, with an IC_50_ of 27.32 μg/mL (Figure 1c, Table 3). Despite this relative resistance, the achieved IC_50_ value remains within clinically relevant ranges and provides important insights into the therapeutic potential and selectivity profile of McEO. Our IC_50_ of 27.32 μg/mL is remarkably competitive compared to other essential oils tested against Raji cells. *Baccharis milleflora* essential oil showed IC_50_ values of 20.07–39.15 μg/mL against the same cell line [42], indicating that our McEO performs well within this therapeutic class. However, some essential oils demonstrate exceptional potency against Raji cells, such as *Lawsonia inermis* (IC_50_ = 0.26 μg/mL) [35] and *Cinnamomum zeylanicum* (IC_50_ = 0.57 μg/mL) [34], suggesting that different phytochemical profiles may confer varying degrees of activity against hematological malignancies. The observed resistance pattern appears to correlate with our network pharmacology findings, which revealed fewer shared molecular targets between McEO components and lymphoma-associated pathways. This mechanistic concordance may suggest intrinsic differences in cellular vulnerability rather than experimental variability. The differential sensitivity between hematological and solid tumor cell lines has been documented extensively, with *Salvia reuteriana* essential oil demonstrating remarkable selectivity for Raji cells (IC_50_ = 21 μg/mL) compared to solid tumor lines (IC_50_ = 156–183 μg/mL) [43]. The selectivity pattern observed (solid tumors > hematological cancers) appears consistent with established literature on terpenoid-rich essential oils and may reflect fundamental differences in membrane composition, drug efflux mechanisms, and survival signaling pathways between cell lineages [44].

### 2.4. Network Pharmacology Analysis

**Figure 2:**
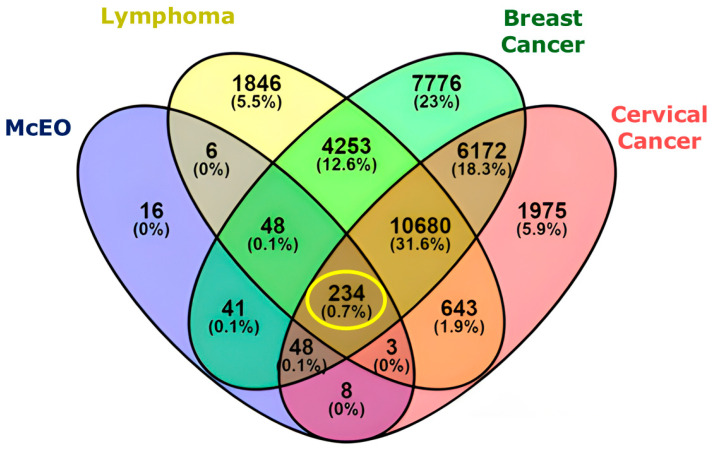
Differential target overlaps between McEO compounds and cancer-associated genes. The Venn diagram illustrates the intersection of predicted protein targets for McEO constituents with genes associated with three cancer types: breast cancer, cervical cancer, and lymphoma. Absolute numbers of shared targets are shown, with corresponding percentages of total identified targets in parentheses. The central overlap (234 targets; 0.7%), highlighted in yellow circle, represents core polypharmacological targets common to all three cancers. Notably, target overlap was highest with breast cancer (23%) and cervical cancer (18.3%), but markedly lower with lymphoma (5.5%).

Network pharmacology was employed to elucidate the multi-target mechanisms underlying *Myrtus communis* essential oil’s (McEO) anticancer activity and to provide a molecular rationale for the cell line-specific sensitivities observed in our MTT assay. This systems biology approach, following methodologies established by Hopkins [20], has proven increasingly valuable for understanding the polypharmacological properties of essential oils in cancer research, with successful applications in studies of *Thymus vulgaris* [45] and *Lavandula* species [46] for breast cancer target identification. The differential cytotoxicity observed across cancer cell lines was explained through target overlap analysis between McEO compounds and cancer-associated genes. As illustrated in Figure 2, this analysis revealed significant disparities that directly correlated with observed cytotoxicity patterns: McEO shared the highest number of targets with breast cancer pathways (23%) and cervical cancer pathways (18.3%), while the overlap with lymphoma-associated targets was substantially lower (5.5%).

This differential targeting pattern provides a molecular explanation for the observed resistance in Raji lymphoma cells and aligns with previous literature demonstrating that *M. communis* essential oil exhibits preferential activity against solid tumors over hematologic malignancies. Supporting this observation, Harassi et al. [47] reported IC_50_ values of approximately 4–6 μg/mL against MCF-7 breast cancer cells, while showing substantially weaker activity against P815 mastocytoma cells. The tissue-specific sensitivity reflects the unique molecular landscapes of different cancers, where the presence or absence of key targets like hormone receptors dictates therapeutic response, a concept supported by numerous studies [48,49].

#### 2.4.1. Hub Compounds and Network Connectivity

**Figure 3:**
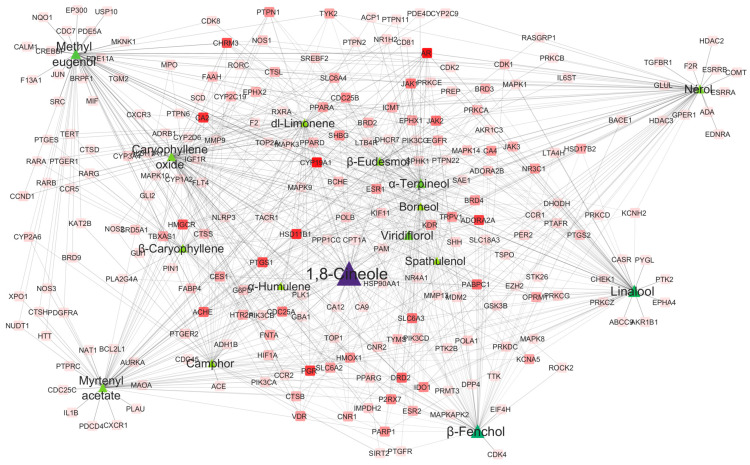
Compound-target interaction network for McEO. Network visualization of pharmacological interactions between McEO’s bioactive compounds and their molecular targets. Green and purple nodes represent bioactive compounds (node size proportional to compound abundance), while red nodes indicate biological targets (color intensity reflects connectivity strength). Key hub compounds including linalool, 1,8-cineole and β-Fenchol demonstrate extensive multi-target interactions, highlighting their potential therapeutic significance. The network topology reveals the polypharmacological nature of McEO, where individual compounds can modulate multiple biological pathways simultaneously.

Topological analysis of the compound-target interaction network identified several key bioactive molecules based on their network centrality metrics, using established centrality measures as described by Chin et al. [50]. The compound-target interactions are visualized in Figure 3 the centrality metrics are detailed in Supplementary Table S1.

Linalool and nerol demonstrated the highest network connectivity, both achieving Maximal Clique Centrality (MCC) scores of 62 and identical closeness centrality values of 0.4102. These monoterpenes occupy central positions within densely connected network clusters, indicating their capacity to simultaneously influence multiple biological targets and coordinate diverse cellular processes [51]. This hub compound behavior aligns with previous studies demonstrating linalool’s multi-target activity across different essential oils. In *Rhododendron anthopogon* oil, linalool acts on TNF-α and EGFR pathways [52], while in *Jasminum sambac*, it exhibits high binding affinity for ESR1, PGR, and CYP19A1 [53]. Our network analysis of McEO reveals similar multi-target engagement patterns, with linalool showing significant interactions with hormone-related receptors and inflammatory mediators, consistent with these published findings. The presence of 1,8-cineole as a central compound in our network analysis is particularly noteworthy when compared to its role in other essential oils. In *Melaleuca quinquenervia* leaf essential oil, 1,8-cineole comprised 31.57% of the total composition and demonstrated selective cytotoxicity against A549 lung cancer cells [54]. While 1,8-cineole showed modest direct binding scores in molecular docking studies, its high abundance and network centrality suggest important adjuvant and synergistic roles in the overall therapeutic effect, similar to our observations in McEO. This pattern supports the concept that major constituents may contribute to therapeutic efficacy through network effects rather than direct target binding alone. In contrast, methyleugenol exhibited the highest betweenness centrality (0.1674), positioning it as a critical “molecular bridge” connecting disparate biological pathways. This phenylpropanoid appears to link hormone signaling pathways, such as ESR1-mediated estrogen signaling, with apoptosis regulation mechanisms, including Mcl-1-mediated survival pathways. Its bridging function is consistent with reported modulation of the mTOR/PI3K/Akt survival pathway and synergistic effects with cisplatin in cervical cancer cells [55]. Supporting this observation, ref. [56] demonstrated that methyl eugenol combined with cisplatin enhanced anticancer activity in HeLa cervical cancer cells, confirming methyleugenol’s role as a chemosensitizer and network bridge compound. However, recent network toxicology studies by [57] revealed that chronic methyl eugenol exposure may promote hepatocellular carcinoma through cell cycle dysregulation, involving upregulation of mitotic kinases (AURKB, PLK1) and cyclin-dependent kinases (CDK1/CCNB1). This finding highlights the importance of dose-dependent therapeutic windows and careful risk-benefit assessment in essential oil applications, particularly when methyleugenol serves as a network hub compound. Additional compounds demonstrated significant network importance: β-Fenchol (MCC = 62, betweenness centrality = 0.0885), β-Eudesmol (MCC = 59), and dl-Limonene (MCC = 58). Topological coefficients ranged from 0.2309 to 0.4522, with Viridiflorol exhibited the highest value (0.4522), indicating its role in maintaining local network cohesion.

#### 2.4.2. Protein Target Network Analysis

**Figure 4:**
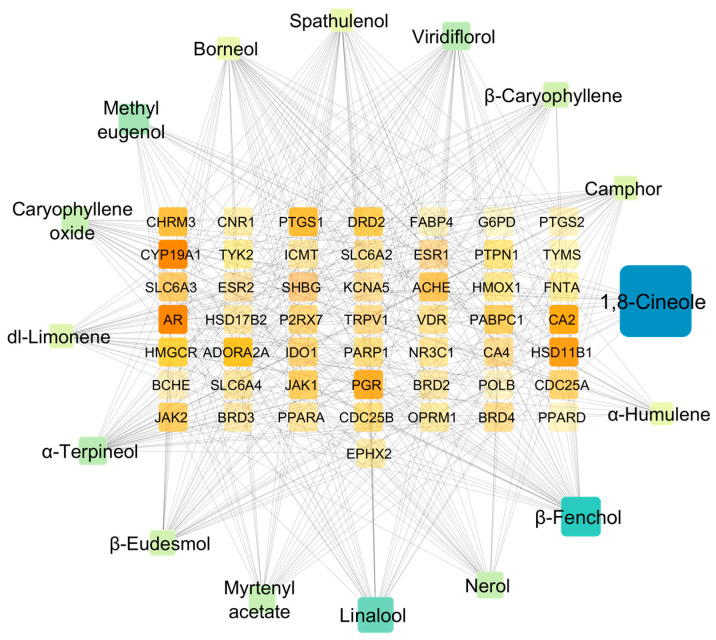
Network centrality metrics of key protein targets. Network of the Main Compounds and Protein Targets Associated with McEO. The figure shows a network of the 50 most important protein targets identified during the analysis, with the active compounds of Myrtle essential oil (green or blue in color, where the size indicate the abundance of the compound in MCEO) interacting with these target genes (orange, where the more orange the color indicated, the more concatenating edge with that node). The connections indicate potentially significant interactions between the compounds and targets, thus contributing to the therapeutic effect of the essential oil.

Protein target analysis identified several hub targets based on their network connectivity metrics. Carbonic Anhydrase II (CA2) demonstrated the highest connectivity with 110 network connections, indicating its central role as a target frequently overexpressed in hypoxic tumor environments (Figure 4; Table S2).

Inhibition of CA2 disrupts cellular pH regulation, exploiting a well-characterized vulnerability of cancer cells [58]. This target’s importance is consistent with findings in other essential oil studies, where CA2 frequently appears as a high-priority target in anticancer networks. Additional targets with 110 connections included Progesterone Receptor (PGR), Sex Hormone-Binding Globulin (SHBG), Acetylcholinesterase (ACHE), and Muscarinic Acetylcholine Receptor M3 (CHRM3), forming a cluster of hormone-related and neurotransmitter targets. The prominence of hormone receptors aligns with findings from MQLEO and thyme oil studies, where ESR1 consistently emerged as a top target [45,54].

Prostaglandin-Endoperoxide Synthase emerged as a key inflammatory target with 100 network connections, directly linking McEO’s anticancer activity to anti-inflammatory mechanisms and suggesting potential benefits in inflammation-driven carcinogenesis [59]. This finding is consistent with research on *Cinnamomum zeylanicum* essential oil, where PTGS was identified as a core target, and benzyl benzoate showed significant binding affinity, contributing to anti-inflammatory effects through reduced PGE2, IL-6, IL-1β, and TNF-α production in LPS-induced RAW macrophages [60], further emphasizing essential oil’s multi-system therapeutic approach. The network also revealed the significant targeting of neurotransmitter systems, with A2A adenosine receptor and D2 dopamine receptor showing approximately 90 connections each. These hub proteins highlight McEO’s comprehensive influence on endocrine, neurotransmitter, and inflammation pathways, providing a molecular framework for understanding its broad cancer-inhibitory actions. The multi-system approach observed in McEO mirrors findings from other essential oils, such as the comprehensive anti-inflammatory mechanism of *Cinnamomum zeylanicum* oil, which involves multi-target modulation of TNF, TLR4, and NFκB pathways, TLR4/NF-κB pathway suppression leading to decreased pro-inflammatory cytokines, and antioxidant activity that mitigates ROS-mediated inflammation [60]. The network-based identification of hub compounds and their bridging functions provides a rational basis for understanding how essential oils can achieve therapeutic efficacy through coordinated multi-target effects rather than single-target inhibition.

#### 2.4.3. Protein–Protein Interaction (PPI) and Functional Enrichment Analysis

**Figure 5:**
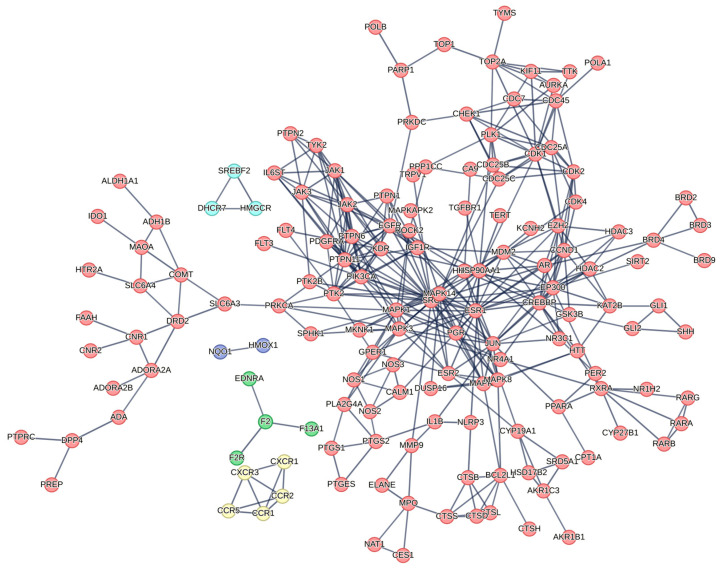
Protein–protein interaction (PPI) network of McEO targets. Protein–protein interaction (PPI) network associated with the antiproliferative effects of McEO. The network illustrates the interactions between proteins, with nodes representing proteins and edges representing their interactions. Functional clusters are grouped according to the biological processes they regulate.

The protein–protein interaction (PPI) network associated with McEO antiproliferative effects comprised 216 nodes and 401 edges, exhibiting an average node degree of 3.71 and a mean local clustering coefficient of 0.433, indicating significant protein interconnectivity (Figure 5). K-means clustering analysis identified five functionally distinct modules that collectively demonstrate McEO’s polypharmacological targeting strategy.

The largest cluster (Cluster 1, 137 genes) encompassed cancer pathways, cellular hormone response mechanisms, and tyrosine kinase receptor signaling, suggesting primary involvement in oncogenic regulation and hormonal modulation. Smaller specialized clusters included immune surveillance mechanisms through dendritic cell chemotaxis and C-type chemokine receptors (Cluster 2, 5 genes), hemostatic regulation via fibrin clot formation pathways (Cluster 3, 4 genes), cholesterol biosynthesis affecting membrane integrity (Cluster 4, 3 genes), and antioxidant enzyme regulation through NFE2L2 signaling (Cluster 5, 2 genes).

**Figure 6:**
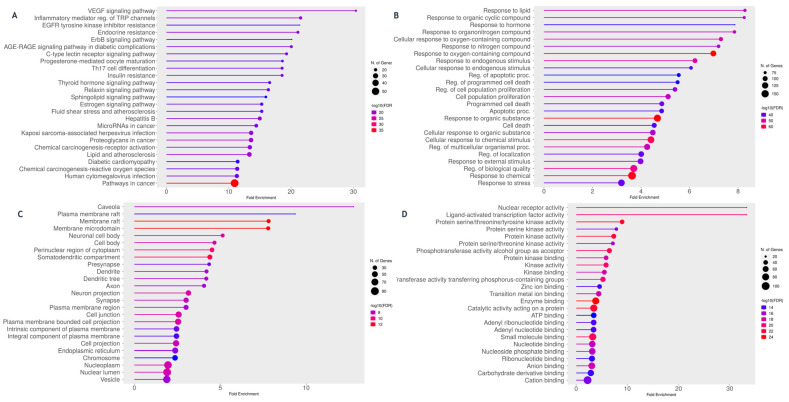
Functional enrichment analysis of McEO target genes. Functional enrichment analysis of genes associated with the antiproliferative effects of McEO. (**A**) KEGG signaling pathway enrichment, (**B**) GO biological processes, (**C**) GO cellular components, and (**D**) GO molecular functions.

Functional enrichment analysis of the target genes revealed several significantly enriched signaling pathways and biological processes (Figure 6).

KEGG pathway analysis revealed the VEGF signaling pathway as the most significantly enriched target (enrichment factor: 29.7), indicating McEO’s anti-angiogenic potential in disrupting tumor vascularization (Figure 6A). Notably, pathways associated with endocrine resistance and EGFR tyrosine kinase inhibitor resistance demonstrated substantial enrichment, suggesting therapeutic utility against treatment-refractory cancers. The AGE-RAGE pathway enrichment in diabetic complications indicates potential applications beyond oncology. Gene Ontology biological process analysis highlighted cellular responses to lipids, organic cyclic compounds, and organonitrogen compounds, demonstrating McEO’s capacity to modulate diverse environmental and metabolic stimuli (Figure 6B). Cellular component analysis revealed preferential targeting of membrane microdomains, including caveolae, lipid rafts, and specialized membrane structures, suggesting bioactive compounds influence cellular signaling through membrane organization modulation (Figure 6C). Additional enrichment in neuronal cell bodies and perinuclear cytoplasmic regions indicates effects on neuronal function and gene expression regulation. Molecular function analysis demonstrated remarkable enrichment in nuclear receptor activity and ligand-activated transcription factors, positioning McEO compounds as gene expression modulators (Figure 6D). Significant protein kinase activity enrichment further supports involvement in intracellular signaling cascade regulation.

### 2.5. Molecular Docking

To evaluate the therapeutic potential of McEO constituents against cancer-relevant targets, comprehensive molecular docking studies were performed using a curated library of phytochemicals against eight oncogenic proteins: Poly(ADP-ribose) polymerase 1 (PARP1), Myeloid cell leukemia-1 (Mcl-1), Cyclin-dependent kinase 6 (CDK6), Phosphoinositide 3-kinase gamma (PI3Kγ), Sirtuin 2 (Sirt2), Estrogen Receptor Alpha (ERα), Heat Shock Protein 90 (HSP90), and Androgen Receptor (AR). Binding affinities were quantified using XP GScore values, with more negative scores indicating stronger predicted binding interactions. All binding affinities were benchmarked against cognate reference ligands for each target (Table 4). Subsequently, 100 ns all-atom molecular dynamics simulations were conducted on four lead complexes to assess the temporal stability and validate the most promising docking predictions.

The docking analysis revealed distinct patterns of target selectivity and binding affinity across all tested proteins, with sesquiterpene alcohols consistently demonstrating superior performance. Spathulenol exhibited exceptional predicted affinity for AR (XP GScore: −9.650 kcal/mol), nearly matching the binding strength of the reference antagonist (−9.721 kcal/mol). This remarkable binding affinity suggests significant therapeutic potential for hormone-dependent cancers, particularly prostate cancer, where AR modulation represents a primary therapeutic strategy. The near-equivalence to the reference AR antagonist is particularly striking given that spathulenol is a natural sesquiterpene alcohol, indicating that nature has evolved highly optimized molecular architectures for protein-ligand recognition that rival synthetic pharmaceutical compounds. Additionally, spathulenol ranked among the top binders for multiple targets, including CDK6 (−7.388 kcal/mol), ERα (−8.650 kcal/mol), and HSP90 (−6.391 kcal/mol), positioning it as a lead multi-target compound within McEO’s constituent profile.

Structure-activity analysis revealed that sesquiterpenes (C15 compounds) consistently outperformed monoterpenes (C10 compounds) across nearly all tested targets. This superior performance can be attributed to their larger molecular scaffolds, which enable more extensive protein-ligand contacts and enhanced shape complementarity within binding pockets [61]. The increased structural complexity of sesquiterpenes allows for greater surface area interactions and multiple contact points with target proteins, aligning with established medicinal chemistry principles and consistent with previous essential oil studies demonstrating enhanced bioactivity of larger terpenes compared to their smaller counterparts hydroxyl moieties facilitate hydrogen bond formation with polar amino acid residues within protein binding sites, providing crucial anchoring points that stabilize protein-ligand complexes [62]. The significance of hydrogen bonding in protein-ligand interactions has been well-established, particularly in epigenetic targets like SIRT2, where such interactions are essential for effective inhibition [63]. This reinforces the hydroxyl group as a key pharmacophore for McEO’s bioactive constituents and suggests that future lead optimization efforts should focus on preserving these critical interactions [64].

**Figure 7:**
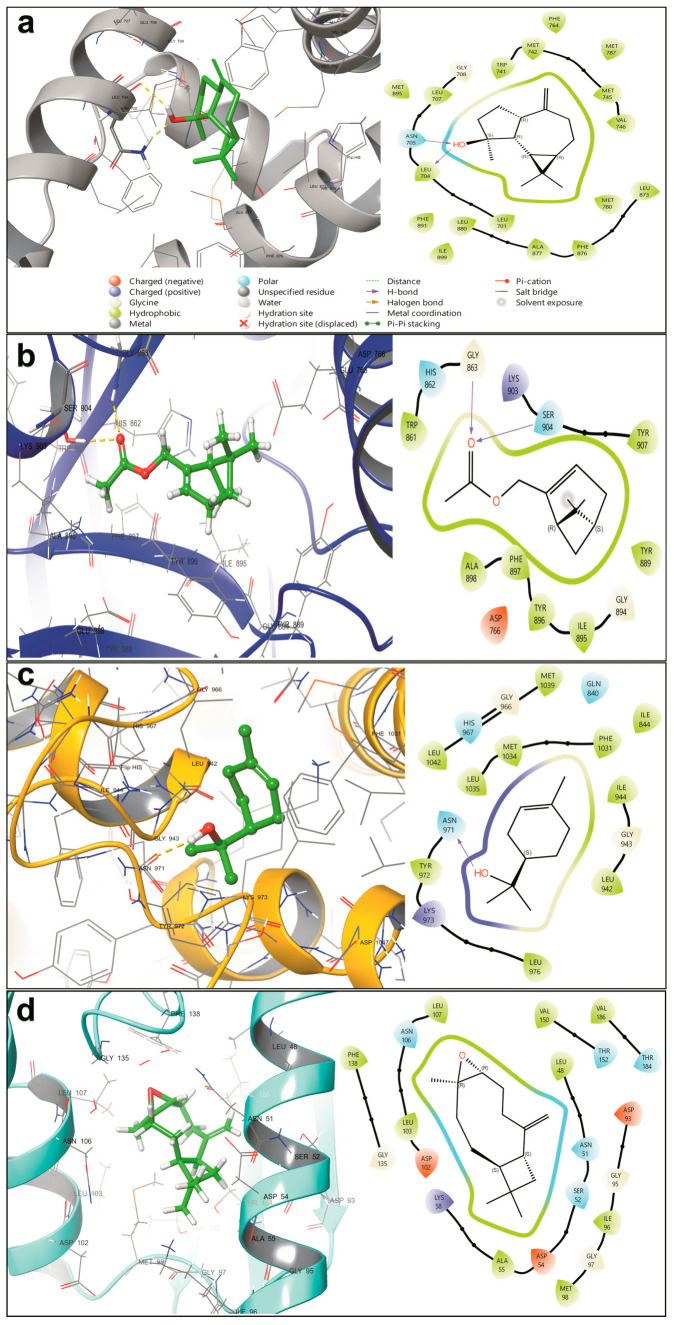
Molecular docking binding modes of lead McEO compounds. Detailed binding interactions of key McEO constituents with cancer targets showing: (**a**) spathulenol-androgen receptor complex, (**b**) myrtenyl acetate-PARP1 complex, (**c**) alpha-terpineol-PI3Kγ complex, and (**d**) β-caryophyllene oxide-HSP90 complex. Protein structures are shown as ribbons with binding pockets highlighted. Ligands are displayed as stick models with key interactions depicted as dashed lines.

Detailed binding mode analysis was performed for the most promising compound-target interactions to elucidate the molecular basis of observed affinities (Figure 7). The spathulenol-androgen receptor interaction was stabilized by two critical hydrogen bonds: a primary contact between the ligand’s hydroxyl group and Asn705 (1.86 Å) and a secondary interaction with the backbone carbonyl of Leu704 (2.17 Å) (Figure 7a). The tricyclic structure demonstrated optimal insertion into the hydrophobic binding pocket, establishing extensive van der Waals contacts with Met742, Met745, Met780, Met895, Leu873, Ala877, Trp741, and Phe876. This binding mode closely mimics that of natural androgens, consistent with established AR ligand recognition patterns where hydrogen bonding with Asn705 represents a key pharmacophoric interaction [65]. The structural complementarity observed suggests that spathulenol may function as an effective AR antagonist, potentially competing with endogenous androgens for binding site occupancy.

The myrtenyl acetate-PARP1 interaction was characterized by the ester functionality forming two specific hydrogen bonds with Gly863 (2.1 Å) and Ser904 (2.3 Å) within the nicotinamide-binding pocket (Figure 7b). Additional π-alkyl interactions between the bicyclic structure and Tyr896 and Tyr907 positioned the ligand for competitive inhibition of NAD+ binding. This binding mode aligns with the structural requirements typically observed for PARP1 inhibitors and suggests a mechanism of synthetic lethality induction, which is particularly relevant given the clinical success of PARP inhibitors in cancer therapy, especially for tumors with deficient DNA repair mechanisms. The identification of myrtenyl acetate as a potential PARP1 inhibitor represents a novel finding that could open new avenues for McEO-based therapeutic development.

The alpha-terpineol-PI3Kγ complex formation was characterized by a critical hydrogen bond between the ligand’s hydroxyl group and Asn971 (1.94 Å), with additional stabilization from water-mediated interactions bridging the ligand to Asp964 and Tyr867 (Figure 7c). The cyclohexene structure showed favorable insertion into the hydrophobic ATP-binding pocket, establishing van der Waals contacts with Leu1035, Met1039, and Lys973. This binding pattern suggests potential modulation of PI3Kγ activity, complementing previously reported anticancer effects of alpha-terpineol through NF-κB suppression [66] and indicating that McEO may simultaneously target multiple nodes within the PI3K/AKT signaling network. The dual targeting of PI3Kγ and NF-κB pathways by alpha-terpineol exemplifies the multi-pathway modulation characteristic of natural product therapeutics.

The β-caryophyllene oxide-HSP90 interaction was characterized primarily by hydrophobic contacts within the N-terminal domain (Figure 7d). The rigid bicyclic sesquiterpene epoxide formed hydrophobic interactions with Ala55, Met98, Ile96, Leu103, Val150, and Val186, supplemented by π-alkyl interactions with Phe138 and Trp162. While direct hydrogen bonds were absent, transient water-mediated contacts with Asn106 were occasionally observed. This interaction profile is consistent with the binding modes of other natural products targeting HSP90 and suggests potential modulation of this critical molecular chaperone, which plays essential roles in protein folding and cellular stress responses. The predominantly hydrophobic nature of this interaction may contribute to allosteric modulation rather than direct competitive inhibition, representing an alternative mechanism for HSP90 targeting.

### 2.6. Molecular Dynamics Simulation

To assess the temporal stability of the most promising docking predictions and validate their therapeutic potential under physiological conditions, 100 ns all-atom molecular dynamics simulations were conducted on four lead protein-ligand complexes: spathulenol-AR, myrtenyl acetate-PARP1, alpha-terpineol-PI3Kγ, and β-caryophyllene oxide-HSP90.

**Figure 8:**
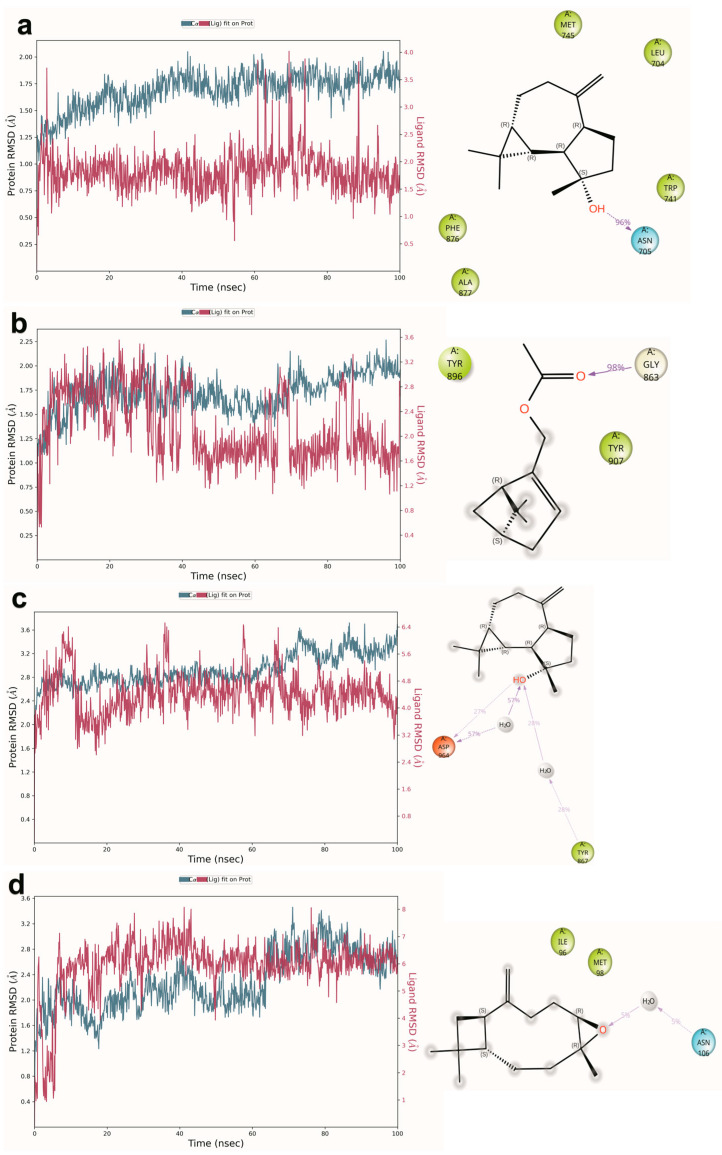
Molecular dynamics simulation trajectories and binding stability analysis. RMSD trajectories and interaction stability profiles for lead McEO compound-target complexes during 100 ns simulations: (**a**) spathulenol-androgen receptor showing exceptional stability, (**b**) myrtenyl acetate-PARP1 demonstrating equilibrium achievement after 60 ns, (**c**) alpha-terpineol-PI3Kγ exhibiting dynamic flexibility, and (**d**) β-caryophyllene oxide-HSP90 displaying high conformational mobility.

The spathulenol-androgen receptor complex demonstrated exceptional thermodynamic stability throughout the simulation period, with minimal protein backbone fluctuations (RMSD 1.5–2.0 Å) indicating optimal ligand accommodation within the binding pocket without significant conformational perturbation (Figure 8a). Most remarkably, the critical hydrogen bond between spathulenol’s hydroxyl group and Asn705 exhibited extraordinary persistence, maintaining 96% occupancy throughout the simulation duration, while stable hydrophobic contacts involving Met745, Leu704, and Trp741 were maintained consistently. This near-permanent interaction network provides compelling computational evidence for spathulenol’s potential as a robust AR antagonist The exceptional stability observed validates the initial docking predictions and indicates that the spathulenol-AR interaction represents a favorable complex that could translate into significant biological activity in experimental systems.

The myrtenyl acetate-PARP1 complex achieved remarkable stability following an initial equilibration period of approximately 60 ns, with protein backbone deviations stabilizing at 1.75–2.00 Å (Figure 8b). The defining interaction between the ligand’s carbonyl oxygen and Gly863 demonstrated exceptional persistence (98% occupancy), effectively anchoring the acetate moiety within the catalytic nicotinamide-binding site. Concurrent π-alkyl interactions with aromatic residues Tyr896 and Tyr907 further stabilized the bicyclic monoterpene core, creating a binding mode consistent with competitive NAD+ inhibition. The sustained binding interactions and structural stability observed throughout the simulation suggest that myrtenyl acetate may function as an effective competitive inhibitor of NAD+ binding, potentially contributing to synthetic lethality induction within McEO’s multi-component activity profile. This finding is particularly significant given the clinical success of PARP inhibitors in treating BRCA-deficient cancers and suggests that McEO may exert anticancer effects through previously uncharacterized DNA repair pathway modulation.

In contrast, the alpha-terpineol-PI3Kγ complex exhibited greater conformational flexibility, reflected in elevated RMSD values for both protein (3.0–3.5 Å) and ligand (3.2–6.4 Å) components (Figure 8c). The direct hydrogen bond with Asp964 showed moderate stability (37% occupancy), but this interaction was significantly augmented by water-mediated hydrogen bonds connecting the ligand to both Asp964 and Tyr867 residues (20–30% occupancy). The cyclohexene structure maintained favorable insertion into the hydrophobic ATP-binding pocket through transient van der Waals contacts with Leu1035, Met1039, and Lys973. This water-mediated stabilization mechanism suggests a more dynamic, yet functionally relevant, binding mode that may contribute to PI3Kγ modulation through allosteric effects rather than direct competitive inhibition, consistent with the complex regulatory mechanisms typical of kinase modulation by natural products.

The β-caryophyllene oxide-HSP90 system represented the most conformationally dynamic complex studied, exhibiting moderate protein stability (RMSD 2.4–2.8 Å) but substantial ligand mobility (RMSD 4.0–8.0 Å) (Figure 8d). Primary stabilizing interactions consisted of sporadic water-mediated hydrogen bonds with Asn106, while hydrophobic residues Ile96 and Met98 provided transient stabilization through van der Waals contacts. The high ligand flexibility observed suggests either a transient binding mode or potential allosteric modulation mechanism rather than direct competitive inhibition, consistent with the complex pharmacological profiles often exhibited by sesquiterpene natural products targeting molecular chaperones. This dynamic behavior is characteristic of natural product interactions with HSP90, where conformational flexibility may contribute to allosteric regulation of chaperone function rather than direct competitive inhibition of ATP binding, potentially offering advantages in terms of selectivity and reduced toxicity compared to direct HSP90 inhibitors.

The comprehensive molecular docking and dynamics analyses provide mechanistic insights into McEO’s observed broad-spectrum antiproliferative effects and differential cancer cell line sensitivities. The computational predictions consistently identified sesquiterpene alcohols, particularly spathulenol, as high-affinity binders across multiple cancer-relevant targets, supporting their role as primary bioactive constituents. The predicted polypharmacological profile, encompassing simultaneous interactions with hormone signaling (AR), DNA repair machinery (PARP1), survival pathways (PI3Kγ), and cell cycle regulators, offers molecular rationale for the multi-target activity observed in cytotoxicity assays. This multi-component approach is consistent with evidence that essential oils achieve enhanced bioactivity through synergistic constituent interactions [67].

The exceptional binding stability demonstrated by spathulenol-AR and myrtenyl acetate-PARP1 complexes suggests these represent the most thermodynamically favorable interactions, aligning with experimental observations of pronounced antiproliferative effects in hormone-dependent cancer cells. Despite representing only 0.29% of total composition, spathulenol’s predicted high-affinity interactions suggest it may function as a key regulatory node, amplifying biological effects through modulation of essential signaling pathways [67]. The identification of multiple binding mechanisms supports the hypothesis that McEO’s therapeutic efficacy derives from synergistic multi-component interactions, potentially offering advantages in circumventing single-target resistance mechanisms common in conventional cancer therapy.

However, these computational predictions require experimental validation through direct binding assays, in vivo animal studies, and systematic toxicity assessment before clinical translation. Future studies should investigate component synergistic interactions and confirm the role of spathulenol despite its low abundance [67].

## 3. Materials and Methods

### 3.1. Plant Material and Essential Oil Extraction

#### 3.1.1. Plant Collection

Dried leaves of *Myrtus communis* were collected during the flowering period (June–July 2023) from the coastal region of Tabarka, Northwestern Tunisia (36.94° N, 8.744786° E). The plant material was harvested in the early morning hours to minimize volatile compound degradation due to heat and sunlight exposure. The collected plant material was air-dried in the shade at room temperature (22 ± 2 °C) for 15 days until the moisture content reached below 10% *w*/*w*, as determined by the loss-on-drying method. Plant identification and authentication were performed by certified botanists Pr. Mohamed Chaieb at the Laboratory of Biodiversity and Ecosystems in Arid Environments (LEBIOMAT), Faculty of Sciences, University of Sfax, Tunisia.

#### 3.1.2. Essential Oil Extraction

McEO was extracted from 100 g of dried leaves using hydrodistillation in a Clevenger-type apparatus. The plant material was immersed in 500 mL of distilled water and subjected to hydrodistillation for 3 h at atmospheric pressure. The distillation temperature was carefully controlled to maintain gentle boiling conditions (50 °C) to prevent thermal degradation of volatile compounds. The collected distillate was dried over anhydrous sodium sulfate (Na_2_SO_4_) to remove residual water traces. The essential oil was separated from the aqueous phase using liquid–liquid extraction with n-hexane (HPLC grade, Sigma-Aldrich, St. Louis, MO, USA) and concentrated using a rotary evaporator (Heidolph Laborota 4000, Schwabach, Germany) under reduced pressure (−0.8 bar) at 35 °C to preserve heat-sensitive compounds. The pure EO was collected and stored in amber glass vials sealed with PTFE-lined caps at 4 °C in darkness until analysis [35].

The extraction yield was calculated using the following formula:

$$\mathrm{Yield}\;\left(\%\right)=\left(\frac{\mathrm{Mass}\;\mathrm{of}\;\mathrm{essential}\;\mathrm{oil}\;(g)}{\mathrm{Mass}\;\mathrm{of}\;\mathrm{dry}\;\mathrm{plant}\;\mathrm{material}\;(g)}\right)\times 100$$

All extractions were performed in triplicate, and the average yield was reported with standard deviation.

### 3.2. Gas Chromatography-Mass Spectrometry (GC-MS)

The chemical composition of McEO was determined using an Agilent 6890N gas chromatograph coupled with a 5973 mass selective detector (Agilent Technologies, Palo Alto, CA, USA). The GC was equipped with an HP-5MS fused silica capillary column (30 m × 0.25 mm, 0.25 µm film thickness; Agilent 19091S-433), coated with 5% phenyl methyl siloxane. Samples were diluted 1:10 (*v*/*v*) in analytical-grade n-hexane, and 1.0 µL was injected manually in split mode (split ratio 30:1) at an injector temperature of 250 °C. Helium was used as the carrier gas at a constant flow rate of 0.9 mL/min, with an average linear velocity of 34.6 cm/s and a column head pressure of 6.5 psi. The oven temperature was initially held at 50 °C for 1 min, then increased at 6 °C/min to 250 °C and held for 3 min, resulting in a total run time of 37.3 min. The transfer line temperature was maintained at 280 °C. Mass spectra were acquired in electron impact (EI) mode at 70 eV. Data were collected in full scan mode over a mass range of *m*/*z* 50–550 with a solvent delay of 3.00 min. The ion source and quadrupole temperatures were set at 230 °C and 150 °C, respectively, with a gain factor of 1.0 and an electron multiplier voltage of approximately 1624 V. Compound identification was performed by comparing mass spectra to those in the NIST and Wiley 9th Edition spectral libraries. Additionally, Kovats’ retention indices (RIs) were calculated using a homologous series of n-alkanes (C_8_–C_24_) analyzed under identical chromatographic conditions, following the method described by Kovats [68].

### 3.3. Cell Lines and Culture Conditions

Raji (RRID:CVCL_0511), HeLa (RRID:CVCL_0030), and MCF-7 (RRID: CVCL_0031) cell lines were obtained from the American Type Culture Collection (ATCC, Manassas, VA, USA) through the Institut Pasteur de Tunis [69,70]. The cells were cultured in RPMI-1640 medium (Gibco, Thermo Fisher Scientific, Waltham, MA, USA) supplemented with 10% heat-inactivated fetal bovine serum (FBS) and maintained in a humidified incubator at 37 °C with 5% CO_2_. Adherent cell lines (HeLa and MCF-7) were subcultured every 3–5 days using 0.25% trypsin-EDTA, while the suspension cell line (Raji) was subcultured by centrifugation (1000 rpm for 10 min). Cell viability was routinely assessed using the trypan blue exclusion method [71]. For the cytotoxicity evaluation, cells were seeded in 96-well plates at a density of 2 × 10^5^ cells/mL in a final volume of 200 μL of complete medium per well. Following a 24 h incubation period to allow for cell adherence, the cells were treated for 48 h with serial dilutions of McEO. The concentration ranges were as follows: 0.078 to 5 μg/mL for Raji cells; 0.781 to 50 μg/mL for HeLa cells; and 1.563 to 50 μg/mL for MCF-7 cells. Untreated cells served as negative control, and wells containing only medium were used as a blank. After the treatment period, the cytotoxicity was measured using a 3-(4,5-dimethylthiazol-2-yl)-2,5-diphenyltetrazolium bromide (MTT) colorimetric assay [72]. Briefly, 20 μL of MTT solution (0.5 mg/mL in PBS; Sigma-Aldrich) was added to each well, followed by a 4 h incubation at 37 °C. The culture supernatant was subsequently removed, and the resulting formazan crystals were dissolved by adding 180 μL of a DMSO: methanol (1:1, *v*/*v*) solution and agitating for 15 min at room temperature. Absorbance was measured at 570 nm using a microplate spectrophotometer. The percentage of cytotoxicity was calculated from the absorbance values, where ‘control’ represents untreated cells, using the following formula:

$$Cytotoxicity\;\left(\%\right)=\left(1-\frac{{\mathrm{Absorbance}}_{\mathrm{control}}-{\mathrm{Absorbance}}_{\mathrm{blank}}}{{\mathrm{Absorbance}}_{\mathrm{treated}}-{\mathrm{Absorbance}}_{\mathrm{blank}}}\right)\times 100$$

### 3.4. Antioxidant Activity

The antioxidant capacity of McEO was evaluated using six complementary spectrophotometric assays targeting distinct oxidative mechanisms. The DPPH radical scavenging activity [73], ABTS radical cation scavenging [74], ferric reducing antioxidant power (FRAP) [75], nitric oxide (NO) scavenging [76], hydrogen peroxide (H_2_O_2_) scavenging [77], and superoxide anion (O_2_^•−^) scavenging [78]. This multi-assay approach provided a comprehensive evaluation of both single-electron transfer (SET) and hydrogen atom transfer (HAT) mechanisms relevant to antioxidant activity. All reagents and reference standards were obtained from Sigma-Aldrich (St. Louis, MO, USA). Protocols were carried out as described in the Supplementary Materials S1, with minor modifications. All assays were performed in triplicate using McEO concentrations ranging from 0.0625 to 1 mg/mL.

### 3.5. Computational Analysis: A Network Pharmacology Approach

#### 3.5.1. Compound Identification and Target Prediction

The constituents of *Myrtus communis* essential oil (McEO), as determined by GC–MS, were selected for further analysis. Three-dimensional structures for each compound were obtained by downloading their respective SDF files from PubChem using PubChem’s REST API and PubChemPy leveraging each compound’s PubChem CID (https://pubchem.ncbi.nlm.nih.gov/; accessed on 11 October 2024) [79]. Potential protein targets in Homo sapiens were predicted using the SwissTargetPrediction web server (https://www.swisstargetprediction.ch/; accessed on 20 October 2024) [80], applying a probability threshold of ≥0.7 to ensure high-confidence interactions. For improved coverage, predictions were cross-validated using the STITCH database (http://stitch.embl.de/; accessed on 25 October 2024) [81], with a minimum confidence score of ≥0.4. All predicted targets were mapped to reviewed UniProt entries for standardization.

#### 3.5.2. Collection of Cancer-Associated Genes

To contextualize the predicted targets, cancer-related genes were retrieved from the DisGeNET database (https://www.disgenet.org/; accessed on 25 October 2024) [82]. Using the following Unified Medical Language System (UMLS) Concept Unique Identifiers (CUIs): “Mammary Carcinoma” (C0678222), “Cervical Carcinoma” (C0302592), and “Lymphoma” (C0024299), corresponding to the MCF-7, HeLa, and Raji cell lines, respectively. Genes with a disease association score ≥0.3 were selected. The intersection of these gene sets with the predicted McEO targets was identified using Venn diagram tools (http://bioinformatics.psb.ugent.be/webtools/Venn/; accessed on 27 October 2024).

#### 3.5.3. Network Construction and Topological Analysis

A compound–target interaction network was constructed using Cytoscape v3.9.1, an open-source platform for biological network analysis (RRID:SCR_003032). The network was imported from a CSV file, with compounds as source nodes and proteins as target nodes. Nodes were categorized as “Compound” or “Protein”, and edge weights were assigned based on interaction confidence scores derived from SwissTargetPrediction (probability ≥ 0.7) and STITCH (confidence ≥ 0.4). Visual attributes such as shape, size, and color were customized to distinguish node types and highlight interaction strength. Topological analysis was performed using the CytoHubba Plugin (RRID:SCR_017677) [50], which calculates centrality metrics including degree, betweenness, closeness, and eigenvector centrality. Nodes with degree ≥2× median and betweenness ≥2.5× median were defined as hubs, consistent with common network pharmacology practices. Global network parameters such as network diameter and average shortest path length were calculated using the built-in network analyzer tool. A protein–protein interaction (PPI) network for overlapping targets was constructed by importing data from the STRING v12.0 database [83], restricted to Homo sapiens, with a minimum interaction confidence score of 0.7 and including up to 50 first-shell interactors. The resulting PPI network was then imported into Cytoscape for further hub detection and network analysis using the same CytoHubba and NetworkAnalyzer protocols.

#### 3.5.4. Functional and Pathway Enrichment Analysis

Functional enrichment analysis of the shared targets was performed using ShinyGO v0.80 (http://bioinformatics.sdstate.edu/go/; accessed on 27 October 2024) [84]. Kyoto Encyclopedia of Genes and Genomes (KEGG) pathway analysis included the top 20 pathways with *p*-values < 0.05. Gene Ontology (GO) terms were categorized into Biological Process (BP), Molecular Function (MF), and Cellular Component (CC), selecting the top 25 terms from each domain based on a false discovery rate (FDR) threshold of <0.05.

### 3.6. Molecular Docking

Docking studies were conducted to evaluate the inhibitory potential of McEO constituents against eight cancer-relevant protein targets: PARP1 (PDB ID 4UND), Mcl-1 (5FC4), CDK6 (5L2I), PI3Kγ (5OQ4), Sirt2 (5YQO), ERα (6CHZ), HSP90 (8AGI), and the androgen receptor (8E1A). Each structure was retrieved from the Protein Data Bank [85] and processed using the Protein Preparation Wizard in Schrödinger Suite 2023-1 (RRID:SCR_014879). The McEO ligands were correspondingly prepared using LigPrep with Epik to generate relevant ionized states (pH 7.0 ± 2.0), and both proteins and ligands were minimized with the OPLS4 force field [86]. A receptor grid was defined by the co-crystallized ligand in each target’s active site. The protocol was first validated by re-docking the native ligand, which successfully reproduced the experimental pose (RMSD < 2.0 Å). Docking of the McEO library was then conducted using Glide in eXtra Precision (XP) mode [87,88].

### 3.7. Molecular Dynamics Simulations

To assess the dynamic stability of the highest-scoring docking predictions, 100 ns all-atom MD simulations were performed on four lead complexes: Spathulenol–AR, Myrtenyl Acetate–PARP1, Alpha-Terpineol–PI3Kγ, and β-Caryophyllene Oxide–HSP90. All simulations were run using the Desmond v5.3 package in Schrödinger 2023-1 (RRID:SCR_014575) [89]. The OPLS4 force field was used for all simulations. Each complex was solvated in an orthorhombic box with the TIP3P water model, extending 10 Å beyond the complex in each dimension. The system was neutralized by adding Na^+^ or Cl^−^ counterions, and a salt concentration of 0.15 M NaCl was used to mimic physiological ionic strength. Before the production run, each system was subjected to a default multi-stage relaxation protocol in Desmond. This protocol includes a series of energy minimizations and short-run simulations under different ensembles to gradually relax the system and equilibrate the temperature and pressure. The production MD run was performed for 100 ns in the NPT ensemble at a constant temperature of 310 K and pressure of 1.013 bar. Temperature was regulated with a Nosé–Hoover chain thermostatc [90], and pressure was maintained with a Martyna–Tobias–Klein barostat. Long-range electrostatic interactions were computed using the Particle Mesh Ewald (PME) method, and a 9 Å cutoff was used for short-range interactions. Trajectory analysis was performed using tools within Maestro. The overall stability of the complexes was evaluated by calculating the RMSD of the protein backbone and the ligand. The flexibility of individual residues was assessed by calculating the RMSF. The persistence of key protein-ligand interactions, including hydrogen bonds and hydrophobic contacts, was monitored throughout the simulation, and their occupancy percentages were calculated.

### 3.8. Data Visualization and Statistical Analysis

All in vitro antioxidant and antiproliferative assays were performed in triplicate, and quantitative data are expressed as mean ± standard deviation (SD). Dose–response curves for cytotoxic and antioxidant activities were generated using nonlinear regression with a four-parameter log-logistic model (LL.4), implemented in R software (version 4.3.0) via the ‘drc’ package (RRID:SCR_001905). Half-maximal inhibitory concentration (IC_50_) values, representing the concentration at which 50% inhibition was achieved, were determined from these fitted models. All data visualization, including dose–response curves and error bars representing the standard deviation, was conducted using the ‘ggplot2’ package in R.

## 4. Conclusions

Cancer’s multifactorial complexity demands innovative polypharmacological strategies that can simultaneously target multiple oncogenic pathways, yet conventional single-target approaches continue to dominate therapeutic development. This study establishes that Tunisian *Myrtus communis* essential oil (McEO) functions as a multi-target anticancer system, with sesquiterpene alcohols, particularly spathulenol, viridiflorol, and β-eudesmol, serving as primary bioactive constituents. Through integrated phytochemical, biological, and computational analyses, we demonstrated that these compounds exhibit strong predicted binding affinities to critical cancer-related proteins including androgen receptor (AR), PARP1, and PI3Kγ, providing a molecular basis for observed antiproliferative effects. The pronounced sensitivity of HeLa cervical cancer cells, combined with computational predictions of stable spathulenol-AR interactions, suggests particular therapeutic promise for hormone-dependent malignancies. These bioactive sesquiterpenes may serve as promising scaffolds or building blocks for rational drug development and structural optimization, offering significant advantages over conventional single-target approaches for treatment-resistant cancers. This work bridges traditional ethnopharmacological knowledge with modern analytical methods, providing a framework for investigating complex natural therapeutic systems. Future investigations should focus on experimental validation of protein-compound interactions, structure-activity relationship studies, in vivo efficacy studies, and clinical translation to fully realize the therapeutic potential of McEO and its derived compounds, demonstrating the potential of natural products to provide multi-target cancer therapeutics with improved clinical outcomes.

## Figures and Tables

**Table 1:** Chemical composition of *Myrtus communis* essential oil.

Compound	RT (min)	Percentage (%)	KI (Exp)	KI (Lit)	Chemical Class
dl-Limonene	8.397	1.37	1024	1028	Monoterpene
1,8-Cineole	8.454	38.94	1026	1031	Oxygenated monoterpene
Linalool	10.079	10.65	1095	1098	
Camphor	11.189	1.76	1141	1146	
β-Fenchol	12.294	13.36	1121	1125	
Borneol	13.795	0.62	1165	1169	
α-Terpineol	16.243	4.68	1186	1189	
Nerol	16.608	3.12	1227	1230	
Methyl eugenol	17.083	6.76	1401	1405	Phenylpropanoid
Myrtenyl acetate	18.272	3.52	1324	1326	Oxygenated sesquiterpene
Caryophyllene oxide	19.515	3.58	1581	1583	
Viridiflorol	20.843	4.72	1590	1593	
β-Eudesmol	21.016	2.15	1649	1652	
α-Humulene	23.249	0.21	1452	1455	Sesquiterpene
β-Caryophyllene	28.994	2.30	1417	1419	
Spathulenol	29.061	0.29	1577	1580	Oxygenated sesquiterpene
Total identified		98.03			

RT = retention time; KI (Exp) = experimental Kovats indices; KI (Lit) = literature Kovats indices obtained from the Wiley spectral library collection and NIST library databases with literature references.

**Table 2:** Antioxidant activity profile of McEO.

Assay	McEO IC_50_ (mg/mL)	Reference Control	Control IC_50_
FRAP	0.008 ± 0.001	Vitamin C	<0.019 mg/mL
DPPH	0.138 ± 0.112	BHT	0.041 ± 0.005 mg/mL
ABTS	0.505 ± 0.050	Trolox	<0.1 mg/mL
H_2_O_2_	0.555 ± 0.055	Vitamin C	<0.1 mg/mL
NO	0.582 ± 0.058	Vitamin C	<0.1 mg/mL
O_2_^−^	1.482 ± 0.148	Vitamin C	<0.1 mg/mL

Results expressed as IC_50_ values (mg/mL, mean ± SD) for six antioxidant assays with standard controls.

**Table 4:** Molecular docking scores of McEO compounds against cancer targets.

Target Protein	Compound	XP GScore (kcal/mol)
PARP1 (PDB: 4UND)	Ref [Talazoparib—fluorinated pyrido-phthalazinone]	−12.187
	β-Eudesmol	−5.702
	Myrtenyl acetate	−5.634
	Spathulenol	−5.297
Mcl-1 (PDB: 5FC4)	Ref [6-chloro-*N*-methylsulfonyl indole carboxamide]	−11.651
	Methyleugenol	−6.519
	Limonene	−6.511
	α-Terpineol	−6.126
CDK6 (PDB: 5L2I)	Ref [Palbociclib—pyrido-pyrimidine kinase inhibitor]	−11.303
	Spathulenol	−7.388
	β-Eudesmol	−6.356
	Myrtenyl acetate	−6.251
PI3Kγ (PDB: 5OQ4)	Ref [PQR309—dimorpholinyl-triazine derivative]	−9.218
	α-Terpineol	−6.970
	Spathulenol	−5.941
	Myrtenyl acetate	−5.645
Sirt2 (PDB: 5YQO)	Ref [L5C—pyrazole carboxamide derivative]	−12.818
	Viridiflorol	−9.207
	β-Eudesmol	−8.936
	Spathulenol	−8.649
Erα (PDB: 6CHZ)	Ref [H3B-9224—indazole-phenylbutene derivative]	−14.240
	Spathulenol	−8.650
	Viridiflorol	−8.547
	β-Eudesmol	−8.207
HSP90 (PDB: 8AGI)	Ref [JMC31—triazole carboxamide derivative]	−11.854
	Spathulenol	−6.391
	Viridiflorol	−6.355
	β-Caryophyllene	−6.108
Androgen Receptor (PDB: 8E1A)	Ref [Thiazole-morpholine derivative]	−9.721
	Spathulenol	−9.650
	β-Eudesmol	−8.989
	Viridiflorol	−8.564
	β-Caryophyllene oxide	−7.967

Binding affinities are expressed as XP GScore values (kcal/mol), with more negative scores indicating stronger predicted binding interactions. Only the top three McEO compounds for each target are shown. Reference ligands are co-crystallized inhibitors from respective PDB structures used for docking validation.

## Data Availability

Data is contained within the article and Supplementary Material.

## Supplementary Materials

The following supporting information can be downloaded at: https://www.mdpi.com/article/10.3390/ph18101542/s1, S1: Detailed Antioxidant Assay Protocols; Figure S1: GC-MS chromatogram of McEO; Figure S2: Dose-response curves showing antioxidant activities of *Myrtus communis* essential oil (McEO) in (A) DPPH, (B) ABTS, (C) FRAP, (D) H_2_O_2_, (E) O_2^−^_, and (F) NO assays; Table S1: Network Centrality Metrics of Key McEO Compounds; Table S2: Key Protein Targets of McEO Bioactive Compounds.
