# Supplementary material for: Integrated Network Pharmacology and Molecular Dynamics Reveal Multi-Target Anticancer Mechanisms of Myrtus communis Essential Oils
Source: Pharmaceuticals (Basel). 2025 Oct 13;18(10):1542. doi: 10.3390/ph18101542 (PMC12566675; doi:10.3390/ph18101542)
Supplement: Supplementary file 1 [file pharmaceuticals-18-01542-s001.zip › pharmaceuticals-3901713-supplementary.pdf]

Supplementary Materials:

Integrated Network Pharmacology and Molecular Dynamics Reveal Multi-Target Anticancer Mechanisms of *Myrtus communis* Essential Oil

S1. Detailed Antioxidant Assay Protocols

1. Total Antioxidant Capacity (TAC) – Phosphomolybdenum Method

Reference: Prieto et al., 1999

Principle: Reduction of Mo(VI) to Mo(V) by antioxidants, forming a green phosphate/Mo(V) complex at acidic pH.

Reagents:

- 0.6 M sulfuric acid
- 28 mM sodium phosphate
- 4 mM ammonium molybdate

Procedure:

1. Mix 0.1 mL of EO (0.0625–1 mg/mL) with 1 mL of reagent solution in a test tube.
2. Incubate at 95 °C for 90 min.
3. Cool and measure absorbance at 695 nm.

Result expression: mg ascorbic acid equivalent (AAE) per gram of EO.

2. DPPH Radical Scavenging Assay

Reference: Sharma & Bhat, 2009

Principle: Reduction of DPPH• radical, color change from purple to yellow.

Reagents:

- 0.004% DPPH in methanol
- EO dilutions (0.0625–1 mg/mL)

Procedure:

1. Mix 250 µL of EO with 500 µL DPPH solution.
2. Incubate in the dark at room temperature for 20 min.
3. Measure absorbance at 517 nm.

Positive control: BHT

Formula:

$$PI(\%) = [(A\_control - A\_sample) / A\_control] \times 100$$

3. ABTS Radical Cation Scavenging Assay

Reference: Re et al., 1999

Principle: $ABTS^{•+}$ scavenging reduces absorbance at 734 nm.

Reagents:

- 7 mM ABTS
- 2.45 mM potassium persulfate
- EO dilutions in methanol

Procedure:

1. Mix ABTS with persulfate and incubate 16 h in dark.

2. Dilute to an absorbance of 0.7 ± 0.01 at 734 nm.

3. Mix 100 µL EO with 900 µL $ABTS^{\bullet +}$ solution.

4. Incubate 6 min at 30 °C.

5. Measure absorbance at 734 nm.

Positive control: Trolox

4. Ferric Reducing Antioxidant Power (FRAP)

References: Oyaizu, 1986; Molina-Díaz et al., 1998

Principle: Reduction of ferric complex to ferrous form, measured at 700 nm.

Reagents:

- 0.2 M phosphate buffer (pH 6.6)
- 1% potassium ferricyanide
- 10% trichloroacetic acid (TCA)
- 0.1% $FeCl_3$

Procedure:

1. Mix 250 µL EO + 500 µL buffer + 500 µL ferricyanide.

2. Incubate 20 min at 50 °C.

3. Add 500 µL TCA, centrifuge 10 min.

4. Mix 750 µL supernatant + 750 µL water + 50 µL $FeCl_3$.

5. Measure absorbance at 700 nm.

Positive control: Vitamin C

5. Nitric Oxide (NO) Scavenging Assay

Reference: Marcocci et al., 1994

Principle: SNP generates NO which forms nitrite, detected via Griess reaction.

Reagents:

- 10 mM sodium nitroprusside
- 1% sulfanilamide in 5% phosphoric acid
- 0.1% N-(1-naphthyl)ethylenediamine

Procedure:

1. Mix 2 mL SNP + 250 µL EO, incubate 15 min at 25 °C.

2. Add 0.5 mL reaction mix + 1 mL sulfanilamide → incubate 5 min.

3. Add 1 mL naphthyl-ethylenediamine → incubate 30 min.

4. Measure absorbance at 546 nm.

Positive control: Vitamin C

6. Superoxide Radical Scavenging (NBT Reduction Assay)

Reference: Yagi et al., 2002

Principle: Superoxide reduces NBT to purple formazan.

Reagents:

- 516.12 mM phosphate buffer
- 6.45 mM EDTA
- 0.096 mM NBT
- 3.87×10^{-3} mM riboflavin

Procedure:

1. Prepare reaction mix and add 100 µL EO.
2. Expose to bright light for 10 min.
3. Measure absorbance at 560 nm.

Positive control: Vitamin C

7. Hydrogen Peroxide (H_2O_2) Scavenging

Reference: Ruch et al., 1989

Principle: H_2O_2 absorbance decreases upon neutralization.

Reagents:

- 40 mM H_2O_2 in 0.1 M phosphate buffer (pH 7.4)
- EO dilutions (0.125–1 mg/mL)

Procedure:

1. Mix 2 mL EO with 1.2 mL H_2O_2.
2. Incubate 20 min at room temperature.
3. Measure absorbance at 230 nm.

Positive control: Vitamin C

8. IC_{50} Determination

All assays were performed in triplicate. Percent inhibition values were plotted against EO concentrations. IC_{50} values were calculated using nonlinear regression with a four-parameter log-logistic model (LL.4) in R (v4.3.0) using the drc package. Model fit was confirmed by residual diagnostics and R^2 evaluation.

S2. Supplementary Figures

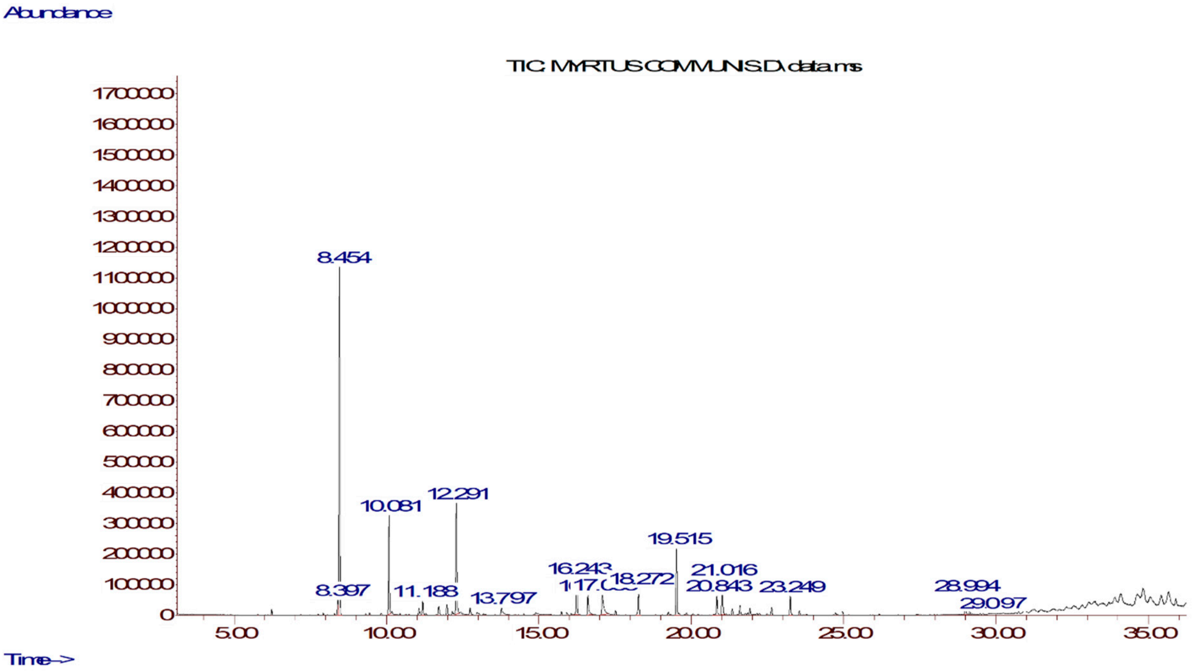

Figure S1. GC-MS chromatogram of McEO

Gas chromatography-mass spectrometry (GC-MS) chromatogram of *Myrtus communis* essential oil (McEO). The chromatogram shows the separation and identification of major bioactive compounds with retention times on the x-axis and relative abundance on the y-axis.

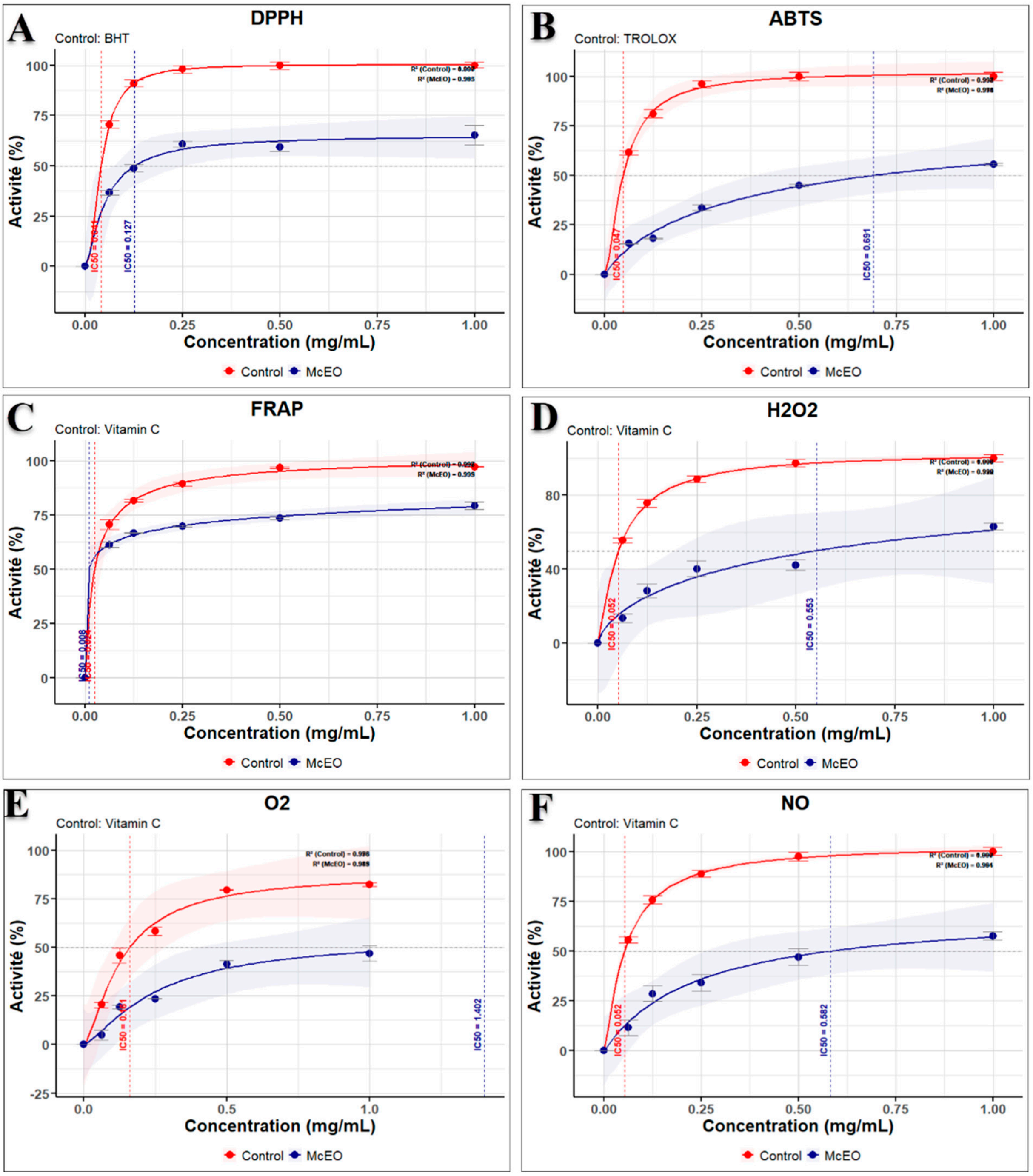

Figure S2. Dose-response curves showing antioxidant activities of *Myrtus communis* essential oil (McEO) in (**A**) DPPH, (**B**) ABTS, (**C**) FRAP, (**D**) H_2O_2, (**E**) O_2^-, and (**F**) NO assays. Blue curves represent McEO, red curves show standard controls. Dashed lines indicate IC_{50} values.

S3. Supplementary Tables

Table S1. Network Centrality Metrics of Key McEO Compounds.

Compounds	MCC Score	Betweenness Centrality	Closeness Centrality	Topological Coefficient
Linalol	62	0.1230	0.4102	0.3129
Nerol	62	0.1441	0.4102	0.2946
β-Fenchol	62	0.0885	0.4102	0.3849
Methyleugenol	56	0.1674	0.4022	0.2309
β-Eudesmol	59	0.0819	0.4062	0.3751
dl-Limonene	58	0.0973	0.4049	0.3287
α-Terpineol	58	0.0861	0.4049	0.3839
Myrtenyl Acetate	58	0.1395	0.4049	0.2942
Caryophyllene Oxide	57	0.1499	0.4035	0.2783
Spathulenol	54	0.0538	0.3983	0.4088
Viridiflorol	52	0.0418	0.3958	0.4522
β-Caryophyllene	51	0.0722	0.3958	0.3254

Table S2. Key Protein Targets of McEO Bioactive Compounds.

Genes	Degree	Betweenness Centrality	Closeness Centrality	Topological Coefficient	Function
AR	150	0.0323	0.4881	0.2391	Androgen Receptor
CYP19A1	150	0.0337	0.4920	0.2354	Aromatase
HSD11B1	120	0.01759	0.4574	0.2634	11β-Hydroxysteroid Dehydrogenase Type 1
CA2	110	0.0177	0.4557	0.2655	Carbonic Anhydrase II
PGR	110	0.0164	0.4490	0.2804	Progesterone Receptor
SHBG	110	0.0081	0.3895	0.3153	Sex Hormone-Binding Globulin
ACHE	100	0.01275	0.4116	0.2767	Acetylcholinesterase
CHRM3	100	0.01462	0.4410	0.2816	Muscarinic Acetylcholine Receptor M3
PTGS1	100	0.01528	0.4288	0.2787	Prostaglandin-Endoperoxide Synthase 1 (COX-1)
SLC6A3	100	0.01085	0.4288	0.2962	Dopamine Transporter
ESR1	100	0.0063	0.3776	0.3362	Estrogen Receptor Alpha
ADORA2A	90	0.01639	0.4458	0.2633	Adenosine A2a Receptor
DRD2	90	0.01276	0.4410	0.2848	Dopaminergic Receptor D2
